# Supplementary material for: Development of a Serum-Free Medium To Aid Large-Scale Production of Mycoplasma-Based Therapies
Source: Microbiol Spectr. 2023 Apr 25;11(3):e04859-22. doi: 10.1128/spectrum.04859-22 (PMC10269708; doi:10.1128/spectrum.04859-22)
Supplement: Supplemental file 1 — Supplemental material. Download spectrum.04859-22-s0001.pdf, PDF file, 1.3 MB [file spectrum.04859-22-s0001.pdf]

SUPPLEMENTARY FIGURES AND TABLES

Figure S1

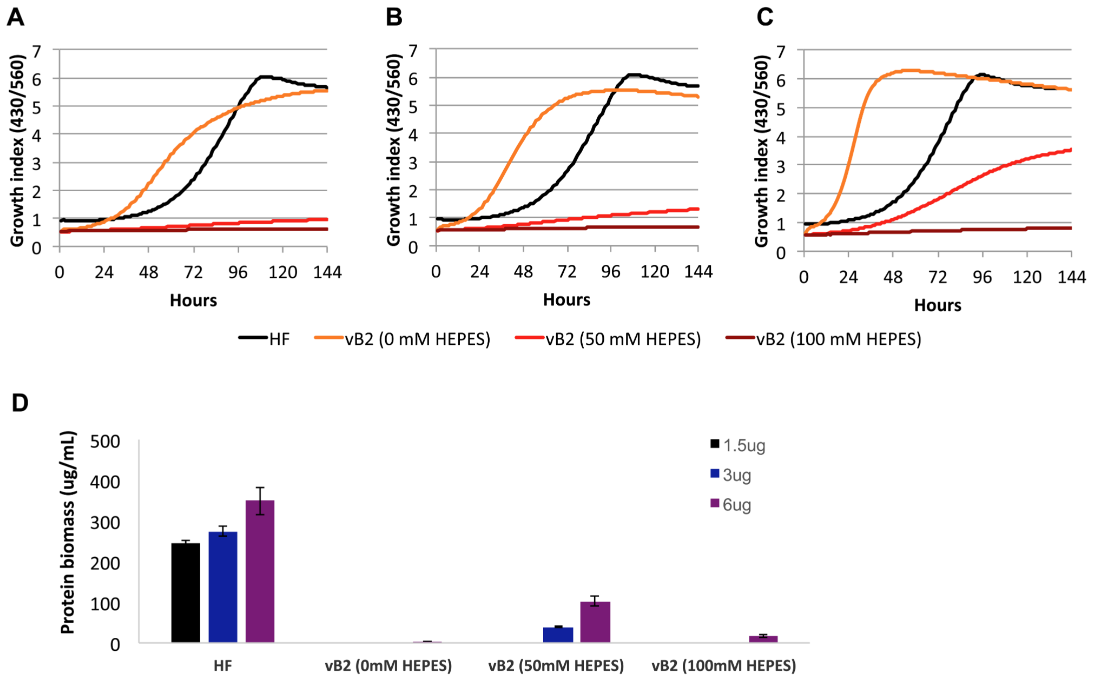

Fig S1. Buffering system optimization for the medium screening.

Growth curve analysis determined by the 430/560 absorbance ratio index method comparing the performance of Hayflick rich medium (HF) and vB2 containing 0mM, 50mM and 100mM HEPES. Cultures were initiated with 1.5µg **(A)**, 3µg **(B)** or 6µg **(C)** of starting inocula. **(D)** Protein biomass yields at the end of the growth curves shown in panels A, B and C. Data represents the mean ± standard error of three biological replicates.

Figure S2

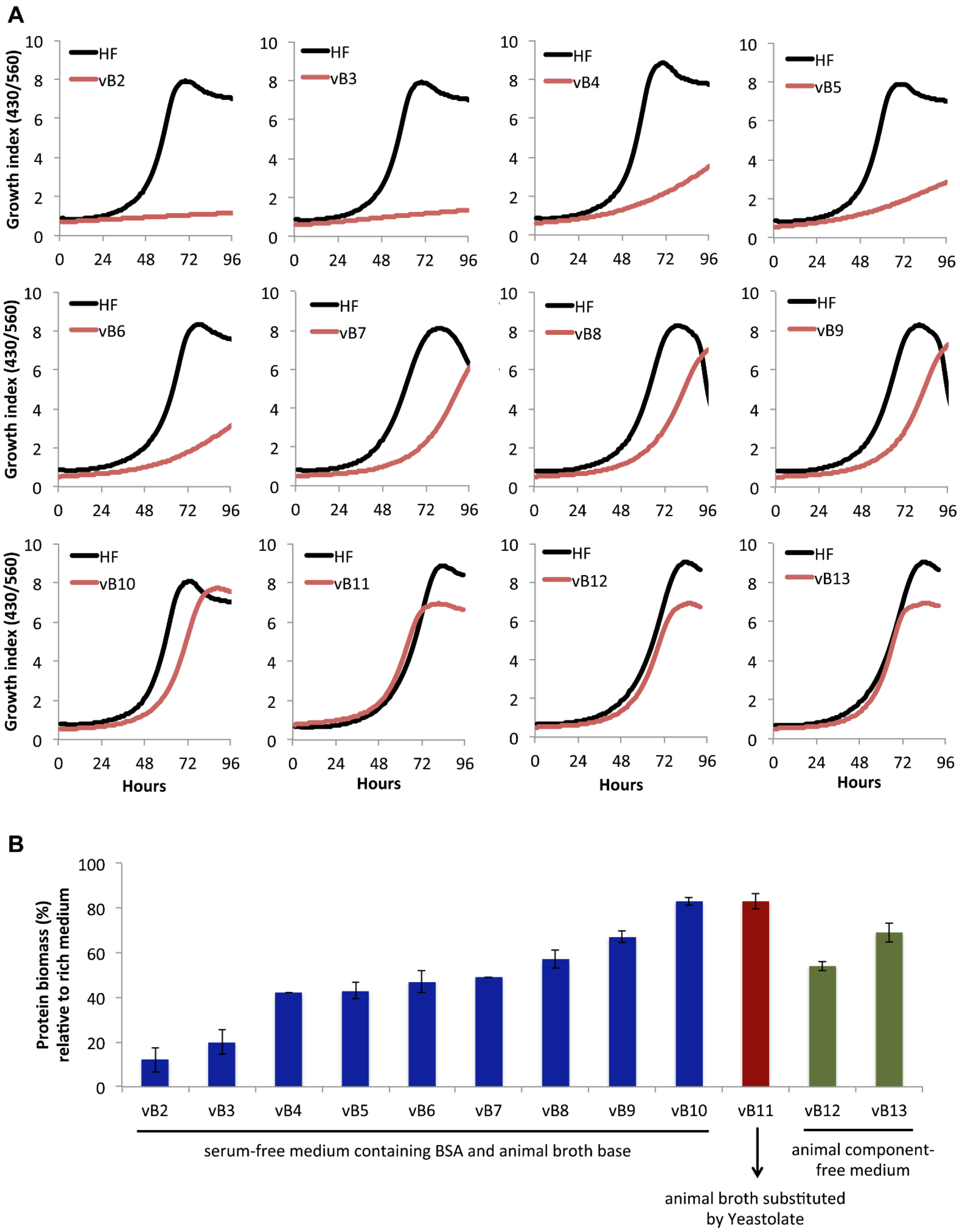

Fig S2. Development and evolution of a serum-free medium for *M. pneumoniae* growth.

(A) Growth curve analyses determined by the metabolic growth index comparing growth performance of different serum-free medium versions to rich medium (Hayflick containing 50mM HEPES).

(B) Percentage of protein biomass yield relative to rich medium (Hayflick) at the end of the growth curves shown in panel A. Data represent the mean ± standard error of two to five biological replicates. The detailed composition of each medium is shown in Table S1.

Figure S3

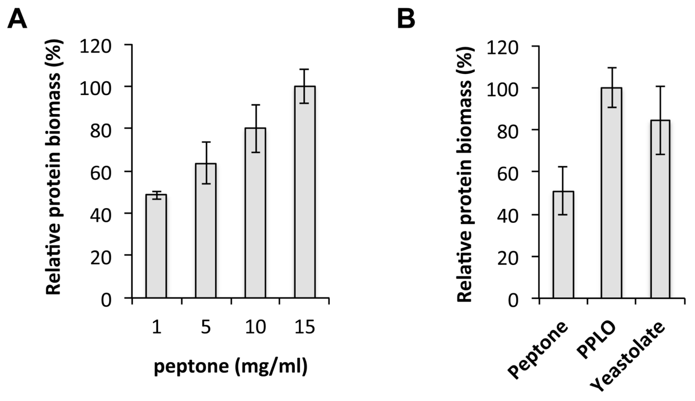

Fig S3. Impact of protein hydrolyzates in medium performance.

(A) Effect of increasing peptone concentration tested in a vB3 formulation.

(B) Comparison of different protein hydrolyzates in vB10 medium performance. Protein biomass yield was assessed in a 96-well plate format culture after 96h of growth. Data represent the mean ± standard error of at least two biological replicates.

Figure S4

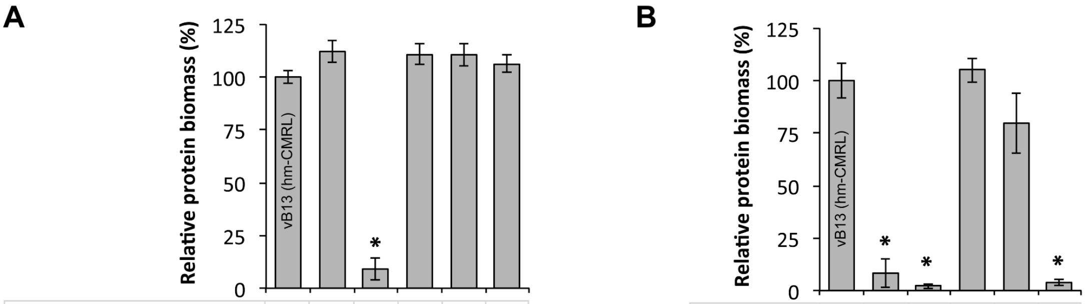

A

group 1: Gly, Hyp, Ala, Arg	+	-	+	+	+	+
group 2: Asp, Cys, CySS, Glu	+	+	-	+	+	+
group 3: His, Iso, Leu, Lys	+	+	+	-	+	+
group 4: Met, Phe, Pro, Ser,	+	+	+	+	-	+
group 5: Thr, Trp, Tyr, Val	+	+	+	+	+	-

B

hm-CMRL	+	-	-	-	-	-
Aspartic acid	-	-	+	-	-	-
Cysteine	-	-	-	+	-	-
Cystine	-	-	-	-	+	-
Glutamic acid	-	-	-	-	-	+

Fig S4. Identification of essential CMRL amino acids.

(A) Identification of groups of amino acids in CMRL that are required for supporting growth in vB13. Data show the relative percentage of protein biomass obtained in medium variants lacking groups of 4 amino acids present in CMRL, and compared to a vB13 medium version, in which commercial CMRL was substituted by a full home-made CMRL (hm-CMRL). Cultures were grown in a 96-well plate format and processed after 96h of culture. The (+) and (-) symbol represent the presence or absence of the indicated group of amino acids present in CMRL. Data represent the mean ± standard error of 3 independent biological replicates. Significance relative to vB13 (hm-CMRL) formulation was assessed by two-sided independent *t*-test (*, *P<0.01*).

(B) Individual assessment of medium performance after substitution of CMRL by each one of the amino acids present in the essential group 2 shown in panel A. Data represent the percentage of protein biomass relative to vB13 (hm-CMRL) as described in panel A, showing that Cysteine or its disulfide form Cystine can replace CMRL.

Figure S5

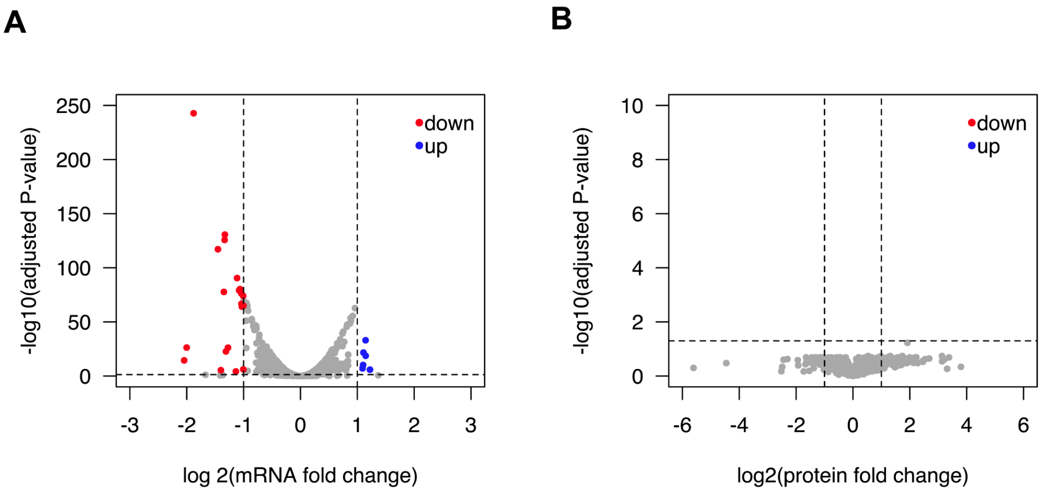

Fig S5. Differential expression analysis of *M. pneumoniae* grown in vB13 serum-free medium and compared to rich medium.

(A) Volcano plot showing transcriptional changes of *M. pneumoniae* grown in vB13 medium. Statistical significance is reported as –log10 of the adjusted p-value after multiple tests correction. Significantly regulated genes (down-regulated in red, and up-regulated in blue) were defined based on a false discovery rate (FDR) of 5% (dashed horizontal line) and a minimum fold change cut-off of 1-log2 (dashed vertical lines). Differential expressed coding-genes are also reported in Table S3.

(B) Volcano plot showing protein changes of *M. pneumoniae* grown in vB13 medium. Statistical significance is reported as for panel A. Data represent the average of two biological replicates. See also Table S4.

Table S1. Detailed media formulation of different versions during *M. pneumoniae* medium optimization

Component	Serum-free medium containing BSA and animal broth base										Animal component-free medium	
	**vB2**	**vB3**	**vB4**	**vB5**	**vB6**	**vB7**	**vB8**	**vB9**	**vB10**	**vB11**	**vB12**	**vB13**
CMRL-1066	2X	1X	1X	1X	0.5X	0.5X	0.5X	0.5X	0.5X	0.5X	0.5X	0.5X
Glucose	0.75%	0.75%	0.75%	0.75%	0.75%	0.75%	0.75%	0.75%	0.75%	0.75%	0.75%	0.75%
HEPES	50mM	50mM	50mM	50mM	50mM	50mM	50mM	50mM	50mM	50mM	50mM	50mM
Ampicillin	100µg/ml	100µg/ml	100µg/ml	100µg/ml	100µg/ml	100µg/ml	100µg/ml	100µg/ml	100µg/ml	100µg/ml	100µg/ml	100µg/ml
Phenol red pH 7	0.0005%	0.0005%	0.0005%	0.0005%	0.0035%	0.0035%	0.0035%	0.0035%	0.0035%	0.0035%	0.0035%	0.0035%
Glycerol	0.005%	0.005%	0.005%	0.005%	0.005%	0.005%	0.025%	0.025%	0.025%	0.025%	0.025%	0.025%
Glutamine	4mM	4mM	2mM	2mM	2mM	2mM	2mM	2mM	2mM	2mM	2mM	2mM
Spermine	10µg/ml	10µg/ml	10µg/ml	10µg/ml	10µg/ml	10µg/ml	10µg/ml	10µg/ml	10µg/ml	10µg/ml	10µg/ml	10µg/ml
Phosphathidylcholine	40µg/ml	40µg/ml	40µg/ml	40µg/ml	40µg/ml	40µg/ml	40µg/ml	40µg/ml	40µg/ml	40µg/ml	40µg/ml	40µg/ml
RNA	1mg/ml	1mg/ml	1mg/ml	1mg/ml	1mg/ml	1mg/ml	1mg/ml	1mg/ml	1mg/ml	1mg/ml	1mg/ml	1mg/ml
Petpone	3mg/ml	3mg/ml	3mg/ml									
PPLO				15mg/ml	15mg/ml	15mg/ml	15mg/ml	15mg/ml	15mg/ml			
Yeastolate										10mg/ml	10mg/ml	10mg/ml
non-lipidated BSA	0.33%	0.33%	0.33%	0.33%	0.33%	0.33%	0.33%	0.33%	0.33%	0.33%		
Cholesterol	33.3µg/ml	33.3µg/ml	33.3µg/ml	33.3µg/ml	33.3µg/ml	33.3µg/ml	33.3µg/ml	33.3µg/ml	33.3µg/ml	33.3µg/ml	30µg/ml	30µg/ml
Palmitic acid	16.6µg/ml	16.6µg/ml	16.6µg/ml	16.6µg/ml	16.6µg/ml	16.6µg/ml	16.6µg/ml	16.6µg/ml	16.6µg/ml	16.6µg/ml		
Oleic acid	20µg/ml	20µg/ml	20µg/ml	20µg/ml	20µg/ml	20µg/ml	20µg/ml	20µg/ml	20µg/ml	20µg/ml		
Thioctic acid						0.2µg/ml	0.2µg/ml	0.2µg/ml	0.2µg/ml	0.2µg/ml	0.2µg/ml	0.2µg/ml
Pyridoxamine							0.5µg/ml	0.5µg/ml	0.5µg/ml	0.5µg/ml	0.5µg/ml	0.5µg/ml
Nicotinic acid								0.5µg/ml	0.5µg/ml	0.5µg/ml	0.5µg/ml	0.5µg/ml
Riboflavin								0.5µg/ml	0.5µg/ml	0.5µg/ml	0.5µg/ml	0.5µg/ml
Choline								0.5µg/ml	0.5µg/ml	0.5µg/ml	0.5µg/ml	0.5µg/ml
Sphingomyelin									40µg/ml	40µg/ml	40µg/ml	40µg/ml
2-Hydroxypropyl)-β-cyclodextrin											5mg/ml	5mg/ml
Tween80											0.00075%	
Tween40											0.0015%	
NaOH (adjust pH7.7)												

Table S2. Detailed list of CMRL constituents present in vB13 medium.

CMRL component	µg/ml in vB13
**Amino acids**	
Glycine	25
Hydroxy L-proline	5
L-Alanine	12.5
L-Arginine hydrochloride	35
L-Aspartic acid	19.5
L-Cysteine	100
L-Cystine	10
L-Glutamic Acid	37.5
L-Histidine hydrochloride-H2O	7.4
L-Isoleucine	10
L-Leucine	30
L-Lysine hydrochloride	31.4
L-Methionine	7.5
L-Phenylalanine	12.5
L-Proline	20
L-Serine	12.5
L-Threonine	15
L-Tryptophan	5
L-Tyrosine	20
L-Valine	12.5
**Vitamins**	
Ascorbic Acid	25
Biotin	0.005
Cholesterol	0.1
Choline chloride	0.25
D-Calcium pantothenate	0.005
Folic Acid	0.005
Niacinamide	0.0125
Nicotinic acid	0.0125
Para-Aminobenzoic Acid	0.025
Pyridoxal hydrochloride	0.0125
Pyridoxine hydrochloride	0.0125
Riboflavin	0.005
Thiamine hydrochloride	0.005
i-Inositol	0.025
**Inorganic salts**	
Calcium Chloride	0.9
Magnesium Sulfate	100
Potassium Chloride	199
Sodium Chloride	58.6
Sodium Phosphate	0.5
**Nucleotide precursors**	
2'Deoxyadenosine	5.4
2'Deoxycytidine	5.8
2'Deoxyguanosine	5.3
5-Methyl-deoxycytidine	0.1
Thymidine	5
Uridine 5'- triphosphate	0.6

CMRL component	μg/ml in vB13
**Other components**	
Co-carboxylase	0.5
Coenzyme A	1.2
D-Glucose (Dextrose)	500
Diphosphopyridine nucleotide (NAD)	3.5
Flavin adenine dinucleotide (FAD)	0.5
Glutathione (reduced)	5
Phenol Red	10
Sodium acetate-3H2O	25
Sodium glucuronate-H2O	2.1
Triphosphopyridine Nucleotide (NADP)	0.5
Tween 80®	2.5

Table S5. Composition of different serum-free medium versions for *M. hyopneumoniae*.

Component	vH1	vH2	vH3	vH4	vH5	vH6
CMRL-1066	0.5X	0.5X	0.5X	0.5X	0.5X	0.5X
Glucose	1.5g/L	1.5g/L	1.5g/L	1.5g/L	1.5g/L	1.5g/L
Sodium pyruvate	2.2g/L	2.2g/L	2.2g/L	2.2g/L	2.2g/L	2.2g/L
Glycerol	0.01%	0.01%	0.01%	0.01%	0.01%	0.01%
Glutamine	2mM	2mM	2mM	2mM	2mM	2mM
Thymidine	40μg/ml	40μg/ml	40μg/ml	40μg/ml	40μg/ml	40μg/ml
Adenosine	10μg/ml	10μg/ml	10μg/ml	10μg/ml	10μg/ml	10μg/ml
Cytidine	10μg/ml	10μg/ml	10μg/ml	10μg/ml	10μg/ml	10μg/ml
Guanosine	10μg/ml	10μg/ml	10μg/ml	10μg/ml	10μg/ml	10μg/ml
Thymine	10μg/ml	10μg/ml	10μg/ml	10μg/ml	10μg/ml	10μg/ml
Yeastolate	10mg/ml	10mg/ml	10mg/ml	7.5mg/ml	7.5mg/ml	7.5mg/ml
Spermine	10μg/ml	10μg/ml	10μg/ml	10μg/ml	10μg/ml	10μg/ml
Thioctic acid	0.2μg/ml	0.2μg/ml	0.2μg/ml	0.2μg/ml	0.2μg/ml	0.2μg/ml
Pyridoxamine	0.5μg/ml	0.5μg/ml	0.5μg/ml	0.5μg/ml	0.5μg/ml	
Nicotinic acid	0.5μg/ml	0.5μg/ml	0.5μg/ml	0.5μg/ml	0.5μg/ml	
Riboflavin	0.5μg/ml	0.5μg/ml	0.5μg/ml	0.5μg/ml	0.5μg/ml	
Choline	0.5μg/ml	0.5μg/ml	0.5μg/ml	0.5μg/ml	0.5μg/ml	
non-lipidated BSA		0.5%	0.5%	0.5%	0.5%	0.5%
Cholesterol	30μg/ml	30μg/ml	30μg/ml	30μg/ml	30μg/ml	30μg/ml
Sphingomyelin	40μg/ml	40μg/ml	10μg/ml	10μg/ml	10μg/ml	10μg/ml
Phosphatidylcholine	40μg/ml	40μg/ml	40μg/ml	40μg/ml	40μg/ml	40μg/ml
PG [(a)]					10μg/ml	10μg/ml
DPPC [(b)]					20μg/ml	20μg/ml
Ampicillin	100μg/ml	100μg/ml	100μg/ml	100μg/ml	100μg/ml	100μg/ml
Phenol red pH 7	0.0035%	0.0035%	0.0035%	0.0035%	0.0035%	0.0035%
NaOH (pH7.7)						

[(a)] PG (L-α-Phosphatidyl-DL-glycerol)
[(b)] DPPC (1,2-Dipalmitoyl-sn-glycero-3-phosphocholine)
